# Editorial: Community series in progress of allo- and xeno-transplantation facilitating the initial xeno-kidney and islet clinical trials, volume II

**DOI:** 10.3389/fimmu.2024.1439832

**Published:** 2024-06-13

**Authors:** Lisha Mou, Burcin Ekser

**Affiliations:** ^1^ Department of Endocrinology, Institute of Translational Medicine, The First Affiliated Hospital of Shenzhen University, Shenzhen Second People’s Hospital, Shenzhen, China; ^2^ MetaLife Lab, Shenzhen Institute of Translational Medicine, Health Science Center, The First Affiliated Hospital of Shenzhen University, Shenzhen Second People’s Hospital, Shenzhen, China; ^3^ Division of Transplant Surgery, Department of Surgery, Indiana University School of Medicine, Indianapolis, IN, United States

**Keywords:** xenotransplantation, islet transplantation, kidney transplantation, genetically modified porcine endothelial cells, PD-L1 overexpression, genetic modifications in pigs, microenvironment in transplantation, hepatocyte microencapsulation

The advent of xenotransplantation has ushered in a new era of possibilities in addressing the severe organ shortage crisis. The current Research Topic in this volume of “Progress of Allo- and Xeno-transplantation Facilitating the Initial Xeno-Kidney and Islet Clinical Trials” offers a collection of pioneering studies that explore various facets of xenotransplantation, particularly focusing on kidney and islet transplantation. This editorial provides an overview of the contributing articles, highlighting their key findings and placing them within the broader context of transplantation research.

## 1 Challenges and opportunities in the islet transplantation microenvironment

Chen et al. presented a comprehensive summary of the challenges and opportunities in the islet transplantation microenvironment. Their study underscores the importance of the microenvironment in determining transplantation outcomes and offers insights into potential strategies to improve islet graft survival with a focus on inflammatory cytokines, immune cells, and vascular endothelial cells.

## 2 Pancreatic islet transplantation: current advances and challenges

In a similar topic but with a different approach, Wang et al. provided a thorough review of the current advances and challenges in pancreatic islet transplantation. They discuss the issues related to islet sourcing, transplantation sites, and immune rejection. Their study highlights the feasibility of inducing stem cells to differentiate into β-like cells *in vitro* and explores the potential of porcine islets in addressing the shortage of islet donors.

## 3 Co-expression of HLA-E and HLA-G on genetically modified porcine endothelial cells

To improve the outcomes in xenotransplantation, Cross-Najafi et al. investigated the co-expression of HLA-E and HLA-G on genetically modified porcine endothelial cells. Their findings demonstrated that the co-expression of HLA-E and HLA-G can significantly attenuate human NK cell-mediated degranulation, shedding light on more successful xenotransplantation outcomes.

## 4 Combined islet and kidney xenotransplantation for diabetic nephropathy

A mini-review by Eisenson et al. provided an update on the ongoing research in combined islet and kidney xenotransplantation for diabetic nephropathy. The authors highlighted the potential of this dual approach in addressing both diabetes and kidney failure, showcasing promising preliminary results with evidence from the published literature.

## 5 Human PD-L1 overexpression in porcine kidneys

In original research by Schmalkuche et al., the effect of human PD-L1 overexpression in porcine kidneys has been explored. This interesting study showed that human PD-L1 genetic modification can reduce xenogeneic human T-cell immune responses, thus enhancing the viability of porcine kidneys in case of clinical xenotransplantation.

## 6 Genetically modified pigs targeting complement activation

Sun et al. discussed the cutting-edge genetic modifications in pigs aimed at targeting complement activation, which has been a major barrier in xenotransplantation. Their study provides valuable insights into the genetic engineering techniques that can mitigate immune rejection.

## 7 Microenvironment and survival in kidney transplantation

Huang et al. conducted a bibliometric analysis to examine the relationship between the microenvironment and survival in kidney transplantation. Their analysis identifies key trends and research hotspots, offering a roadmap for future studies in this critical area.

## 8 Advances in hepatocyte microencapsulation

Wang et al. reviewed the advances in hepatocyte microencapsulation, focusing on selecting materials and preservation methods. Their comprehensive review highlights the progress made in enhancing the viability and functionality of encapsulated hepatocytes for transplantation.

## 9 Developments in kidney xenotransplantation

Xu and He presented a detailed overview of the developments in kidney xenotransplantation. Their study highlights the significant strides made in genetic modifications and immunosuppressive protocols, which are crucial for the success of clinical xenotransplantation.

## 10 Ethical and legislative advances in xenotransplantation

With his expertise over decades, Hawthorne discussed the ethical and legislative advances in xenotransplantation, with a main focus on cardiac xenotransplants. His paper emphasizes the importance of ethical considerations and regulatory frameworks in advancing xenotransplantation to clinical practice in a safe manner.

## 11 Anesthesia and surgery in kidney xenotransplantation

Zhang et al. explored the role of anesthesia and surgical techniques in advancing kidney xenotransplantation to clinical practice. Their study bridges the gap between preclinical and clinical practices, offering insights into optimizing surgical outcomes.

The collection of articles by many experts in this volume provides a comprehensive overview of the current state of xenotransplantation research. Each study contributes valuable knowledge to the field, addressing various challenges and proposing innovative solutions.

As we move closer to clinical reality with limited cases happening all over the world, it is imperative to continue interdisciplinary collaboration and rigorous research to overcome the remaining hurdles in xenotransplantation worldwide. This collective effort will lead to successful clinical trials and ultimately, a wider application of xenotransplantation in addressing the organ shortage crisis globally.

## Author contributions

LM: Writing – original draft. BE: Writing – review & editing.

